# *Kingella kingae* Causing Septic Arthritis of the Knee in an *Immunocompetent* Adult

**DOI:** 10.1155/2015/519190

**Published:** 2015-06-14

**Authors:** J. Ricketts, N. N. T. Rehmatullah, P. Sutton

**Affiliations:** ^1^Department of Trauma and Orthopaedics, Warrington Hospital, Lovely Lane, Warrington WA5 1QG, UK; ^2^Department of Trauma and Orthopaedics, Northern General Hospital, Herries Road, Sheffield S5 7 AU, UK

## Abstract

The bacterium *Kingella kingae* is a species of Gram-negative coccobacillus usually found in the oropharynx. This is an emerging pathogen reported to cause bacteraemia, endocarditis, and osteoarticular infections in children and endocarditis in the immunocompromised adult. However, there are few cases of isolated joint infections reported in the immunocompetent adult. Due to specific isolation techniques required, delay in diagnosis can compromise patient outcome. We report a rare case of septic arthritis of the knee in an immunocompetent adult caused by *K. kingae*.

## 1. Introduction

*Kingella kingae* is a fastidious Gram-negative *β*-haemolytic coccobacillus isolated in the normal respiratory flora in as many as 5–12 per cent of nasal and pharyngeal cultures [1]. First described by King in the 1960s [2] for most of its early history it was considered a rare cause of infection [3]. Over the past 2 decades, an increasing number of reports of septic arthritis, osteomyelitis, and endocarditis have been described secondary to* K. kingae* infection in children [3]. This is now recognised as an emerging pathogen [3] and a common cause of skeletal infections in young children [4, 5]. Improving culture techniques and familiarity with the unique bacteriologic characteristics of this organism have increased the frequency of its detection [6]. In adults, however, far fewer cases have been described. These are commonly opportunistic, presenting almost exclusively in immune-compromised patients. We report a prolonged case of isolated septic knee arthritis secondary to* K. kingae* in an immunocompetent adult. This is only the second such case reported in the literature to the author's knowledge.

## 2. Case Report

A 36-year-old lady presented to the Emergency Department with a five-day history of progressively worsening right knee pain. She denied any trauma and had no other past medical history. Physical examination revealed a mild pyrexia of 38.2°C, a moderate sized effusion, warmth of the joint, tenderness on palpation, and an arc of movement of 20 degrees. Her respiratory, cardiac, abdominal, urological, and neurological systems were normal. Blood tests showed a raised white cell count (WCC) of 17 × 10^9^/L, C-reactive protein (CRP) of 132 mg/L, and erythrocyte sedimentation rate (ESR) of 78 mm/h. Aspiration of the knee joint produced 25 mls of straw-coloured fluid but no organisms or crystals were isolated on microscopy. Blood and urine cultures were unremarkable and the patient underwent an arthroscopic knee washout later that day. Intraoperative samples were negative for organisms and she was discharged pain-free 2 days postoperatively without antibiotics.

Six weeks later, the patient presented again to the Accident and Emergency Department with worsening symptoms and raised inflammatory markers. WCC was again raised at 22.7 × 10^9^/L, CRP at 184 mg/L, and ESR at 83 mm/h. Repeat knee aspiration showed no organisms or crystals. The patient underwent a second arthroscopic washout and was placed on intravenous flucloxacillin and benzylpenicillin (1 gram and 1.2 grams QDS, resp.) for seven days prior to discharge on oral penicillin (1 gram QDS) for a total of 6 weeks.

After further 4 weeks, she re-presented with identical symptoms. Inflammatory markers had not changed since the previous admission. A third knee aspiration produced 85 mls of straw-coloured, turbid fluid that again was not purulent in appearance. This time, some aspirate was placed into standard blood culture bottles (aerobic and anaerobic) in addition to regular sterile containers and microscopy was performed on all 4 samples.

Inoculation of the aerobic blood culture sample for 2 weeks on blood agar in a CO_2_ enriched atmosphere allowed the growth of a Gram-negative coccobacillus. This was identified as* Kingella kingae* (a nonmotile, nonfermenting, weakly oxidase-positive, catalase-negative, and urease-negative organism which produces beta-haemolysis on blood agar) [7] which was sensitive to gentamicin and ciprofloxacin only. The patient underwent an open knee washout and was started on 6 weeks of oral ciprofloxacin. There were no further acute presentations. One year after presentation, the patient complains of some residual pain and a loss of 20 degrees of joint flexion.

## 3. Discussion

*Kingella kingae* is a slow-growing, fastidious Gram-negative member of the HACEK group of microorganisms [7–9], which form part of the normal commensal of the oral and pharyngeal cavities [7]. Over the past decade, it has emerged as a significant pathogen in the paediatric age group primarily causing bacteraemia, endocarditis, and osteoarticular infections. Paediatric osteoarticular infections due to* K. kingae* are commonly preceded by oropharyngeal infection and subsequently are most prevalent during the winter months [1, 3]. Recent evidence in children from clusters of infection in daycase centres worldwide suggests the presence of a “carrier state” [1], and it has been postulated that* K. kingae* present on the oropharynx may penetrate and invade the bloodstream through a damaged mucosal layer [8]. In contrast, presentation in later life occurs almost exclusively in those with significant other predisposing factors. Examples frequently causing immunosuppression include haematological malignancies, acquired immunodeficiency syndrome, systemic lupus erythematosus, diabetes mellitus, rheumatoid arthritis, tumours, and end stage renal disease [10]. Infection involving the bone or joint is rare, more classically causing endocarditis, spondylodiscitis, or bacteraemia [10].

The isolation of* K. kingae* as the sole causative bacterium in an otherwise well adult was extremely unusual. Peak prevalence of invasive infection occurs in 2-year-olds, and this shows an association with the pharyngeal colonisation rate of 10–12 per cent in this group. However, this rapidly declines with age, and immunologically competent adults are rarely carriers [11]. In one 14-year study, 98.6 per cent of all invasive cases caused by* K. kingae* occurred below the age of 4 [11]. The patient in our case had no known close exposure to young children who may have acted as bacterial carriers.

Following an extensive literature review, we identified only 7 case reports of septic arthritis in adults [10, 12–16]. There was only one reported case of isolated infection in an otherwise immunocompetent adult; this also affected the knee [8]. All other cases identified involved patients with significant predisposing factors. Elyès et al. also described an adult who developed endocarditis in addition to septic knee arthritis secondary to* K. kingae* [7].

Our case showed a relapsing course, most likely due to inadequate and untargeted antibiotic treatment (in an otherwise healthy patient) following the second presentation. It is important to note that primary culture on solid mediums frequently fails to isolate this bacterium [17]. Initial Gram staining has been found to be positive in only a small percentage, and the leucocyte value <50,000 WBC/mm^3^ in one-quarter of samples [6, 18]. Contaminants present in purulent synovial fluid itself also exert an inhibitory effect on* K. Kingae* recovery in vitro. The effect of this may be lessened by dilution in large volumes of broth to decrease the concentration of as yet unidentified components and allow for recovery [17, 19, 20]. This can be achieved by placing synovial fluid samples directly into aerobic blood culture bottles which can then be inoculated on blood agar cultures in a CO_2_ enriched atmosphere [7]. In one study, following a mean incubation time of 4 days, this yielded growth in all cases compared to only 8 per cent cultured on traditional solid plates [19].

As an emerging pathogen,* K. kingae* is previously thought to have been mistaken for contaminant or simply misidentified prior to awareness and recognition of the key features required for correct microbial identification [6, 19]. Adding to this* K. kingae* may also appear Gram-positive on staining due to its propensity to retain crystal violet dye [1]. Use of nucleic acid amplification assays (NAAAs) enables exact identification even after the use of empirical antibiotics and can improve the average time to diagnosis from 3-4 days to less than 24 hours [1].

Treatment should be directed according to sensitivities; however,* K. kingae* is commonly susceptible to a range of antibiotics, including *β*-lactams such as penicillin, gentamicin, cephalosporins [3], macrolides, and tetracyclines. Of note, resistance has been shown to vancomycin which may complicate empirical treatment for presumed staphylococcal joint infection. This highlights the importance of early organism identification and targeted antibiotic therapy. Resistance is also reported to erythromycin, clindamycin, trimethoprim, and ciprofloxacin in vitro [6, 21, 22].

To conclude, this is the first reported case of isolated septic arthritis occurring in an immunocompetent adult. We highlight the need for orthopaedic surgeons to be aware of the patient demographics most frequently affected by this rare bacterium and the isolation technique required to obtain an early diagnosis and prevent subsequent joint destruction.
